# The TRPM2 Ion Channel Regulates Inflammatory Functions of Neutrophils During *Listeria monocytogenes* Infection

**DOI:** 10.3389/fimmu.2020.00097

**Published:** 2020-02-04

**Authors:** Frank H. Robledo-Avila, Juan de Dios Ruiz-Rosado, Kenneth L. Brockman, Santiago Partida-Sánchez

**Affiliations:** ^1^Center for Microbial Pathogenesis, The Abigail Wexner Research Institute at Nationwide Children's Hospital, Columbus, OH, United States; ^2^Department of Microbiology and Immunology, Medical College of Wisconsin, Milwaukee, WI, United States; ^3^Department of Pediatrics, College of Medicine, The Ohio State University, Columbus, OH, United States

**Keywords:** TRPM2, *Listeria monocytogenes*, neutrophils, inflammation, systemic inflammation

## Abstract

During infection, phagocytic cells pursue homeostasis in the host via multiple mechanisms that control microbial invasion. Neutrophils respond to infection by exerting a variety of cellular processes, including chemotaxis, activation, phagocytosis, degranulation and the generation of reactive oxygen species (ROS). Calcium (Ca^2+^) signaling and the activation of specific Ca^2+^ channels are required for most antimicrobial effector functions of neutrophils. The transient receptor potential melastatin-2 (TRPM2) cation channel has been proposed to play important roles in modulating Ca^2+^ mobilization and oxidative stress in neutrophils. In the present study, we use a mouse model of *Listeria monocytogenes* infection to define the role of TRPM2 in the regulation of neutrophils' functions during infection. We show that the susceptibility of Trpm2^−/−^ mice to *L. monocytogenes* infection is characterized by increased migration rates of neutrophils and monocytes to the liver and spleen in the first 24 h. During the acute phase of *L. monocytogenes* infection, Trpm2^−/−^ mice developed septic shock, characterized by increased serum levels of TNF-α, IL-6, and IL-10. Furthermore, *in vivo* depletion of neutrophils demonstrated a critical role of these immune cells in regulating acute inflammation in Trpm2^−/−^ infected mice. Gene expression and inflammatory cytokine analyses of infected tissues further confirmed the hyperinflammatory profile of Trpm2^−/−^ neutrophils. Finally, the increased inflammatory properties of Trpm2^−/−^ neutrophils correlated with the dysregulated cytoplasmic concentration of Ca^2+^ and potentiated membrane depolarization, in response to *L. monocytogenes*. In conclusion, our findings suggest that the TRPM2 channel plays critical functional roles in regulating the inflammatory properties of neutrophils and preventing tissue damage during *Listeria* infection.

## Introduction

Polymorphonuclear cells (PMNs), commonly called neutrophils, are the first line of defense of the host against microbial infections (1–3). During an active infection, neutrophils migrate to the site of inflammation following chemotactic gradients of chemokines and pathogen-associated molecular patterns (PAMPs) (4, 5). Neutrophils eliminate pathogens through phagocytosis, granule release or the production of neutrophil extracellular traps (NETs) (1, 6), these mechanisms are in part regulated by mobilization of calcium (Ca^2+^) and the subsequent Ca^2+^ signaling events (7, 8).

Although Ca^2+^ release-activated channels (CRAC) are considered the main ion channels responsible for the regulation of Ca^2+^ entry in immune cells (7, 9), the transient receptor potential (TRP) superfamily have emerged as crucial ion channels that regulate specific cell processes in myeloid cells (10–12). Particularly, the transient receptor potential melastatin 2 (TRPM2), has been proposed to regulate inflammatory responses in myeloid cells (13–16). TRPM2 is a Ca^2+^ permeable, non-selective cation channel, which is activated by ADP-ribose (ADPR), temperature, oxidative stress and Ca^2+^ (17). TRPM2 is highly expressed in dendritic cells, macrophages, and neutrophils (10). Activation of TRPM2 results in transport of Ca^2+^ across the plasma membrane and cytosolic Ca^2+^ release in lysosomes (18). Studies using inflammatory models of TRPM2-genetically deficient mice (Trpm2^−/−^) have revealed the importance of this channel in the immunity of the host (13). A previous study showed that Trpm2^−/−^ mice are susceptible to *L. monocytogenes* infection, presumably through reduced production of IL-12 and IFN-γ, suggesting a defective interplay between innate and adaptive immune responses in the Trpm2^−/−^ mice (19). However, recent work supports the hypothesis that TRPM2 functions as a negative regulator of inflammation. Favoring this premise, Trpm2^−/−^ mice challenged with lipopolysaccharide (LPS) developed greater inflammatory responses, which correlated with a reduced survival rate as compared to WT mice (20). Moreover, Trpm2^−/−^ mice displayed increased vascular damage due to exacerbated migration of neutrophils into the tissue in a model of neutrophil-mediated vascular injury (16). Results from our group also showed that loss of TRPM2 yielded increased production of inflammatory mediators and M1 polarization of macrophages during *H. pylori* infection, which correlated with greater gastric inflammation in chronically *H. pylori*-infected Trpm2^−/−^ mice (14). Yet, the mechanisms by which TRPM2 regulates the development of the inflammatory response during bacterial infections are not fully understood.

Here, we aimed to elucidate the cellular innate inflammatory mechanisms responsible for the increased susceptibility of Trpm2^−/−^ mice to infection with *L. monocytogenes*. We found that lethality in Trpm2^−/−^ mice infected with *L. monocytogenes* is caused by the overwhelming bacterial burden in the liver and spleen, which follows increased neutrophil and monocyte recruitment to the liver. The systemic inflammatory response elicited in the absence of TRPM2 in infected mice culminated in septic shock. Unexpectedly, depletion of neutrophils in Trpm2^−/−^ mice rendered resistance to *L. monocytogenes* infection and a reduced inflammatory cytokine storm in these mice. The highly inflammatory profile of Trpm2^−/−^ neutrophils was further confirmed *in vitro*, linked to augmented Ca^2+^ entry and the enhanced membrane depolarization triggered by bacteria in these cells. Together, our results suggest an essential functional role for TRPM2 channel in the regulation of neutrophils' inflammatory responses that follow bacterial infection.

## Materials and Methods

### Mice Strains

Wild-type (WT) C57BL/6, B6N.129S2-*Ncf1*^*tm*1*Shl*^/J (Nox2^−/−^), and B6.129S4-*Ccr2*^*tm*1*lfc*^/J (Ccr2^−/−^) were originally purchased from Jackson's Laboratory. Trpm2^−/−^ mice were backcrossed for over 10 generations into the C57BL/6J genetic background (21) and were originally donated by Dr. Y. Mori, University of Kyoto, Japan. All animals were bred and maintained in Nationwide Children's Hospital vivarium.

### Bacterial Culture

*Listeria monocytogenes* 10403S and *Listeria monocytogenes* Xen-32 (constitutively bioluminescent) were cultured in Brain-Hearth infusion broth (Difco) at 37°C for 4–6 h, bacterial concentration was adjusted before each experiment based on absorbance at 600 nm.

### *L. monocytogenes* Infection Model

C57BL/6 (WT) and Trpm2^−/−^ mice were intravenously (*i.v*.) infected with 10^4^ colony-forming units (CFU) of *L. monocytogenes*, and the animals were monitored for up to 8 days post-infection (dpi). Some WT or Trpm2^−/−^ mice were *i.v*. injected with 250 μg of anti-Ly6G (1A8) or anti-Gr1 (RB6-8C5) 24 h before infection and 48 h post-infection (hpi). Results were graphed using Kaplan-Meier curves.

### Tracking of *L. monocytogenes* Spreading *in vivo*

WT and Trpm2^−/−^ mice were *i.v*. infected with 10^8^ CFU of *L. monocytogenes* Xen-32 as previously described (22). Mice were anesthetized with isoflurane 4 h post-infection and bacterial dissemination was tracked using Xenogen IVIS Imaging System (Perkin Elmer Inc.). Photons were measured during 1 min exposure by keeping the animals in the ventral position. Following the procedure, mice were euthanized and livers and spleens were collected. Bacterial burden within the infected organs was also measured by a 30 s exposure on the Xenogen IVIS system. Photon emissions were quantified with Living Image software (Caliper Life Science).

### Isolation of Mouse Neutrophils

Mouse bone marrow cells were isolated from femurs and tibiae. Polymorphonuclear cells (PMN) were purified by negative selection (Stemcell Technologies) according to the manufacturer's directions. For inflammatory neutrophils, mice were injected intraperitoneally (*i.p*.) with 1 ml of 4% of thioglycolate, 24 h later peritoneal contents were collected and neutrophils were purified by positive selection using biotinylated anti-Ly-6G (Biolegend) and MACS streptavidin-microbeads (Miltenyi Biotec), following the manufacturer's instructions.

### Quantitation of Total Superoxide Species

A total of 10^5^ neutrophils were seeded into the wells of 96 well black plates and incubated for 10 min at 37°C. Some cells were pretreated with 10^−5^ M Diphenyleneiodonium chloride (DPI) (Tocris Bioscience), for 10 min at 37°C, followed by the addition of 10^−4^ M of luminol (Sigma Aldrich). Cells were incubated for 5 min at 37°C, and then, stimulated with 10^−7^ M of phorbol 12-myristate 13-acetate (PMA) (Acros organics) or *L. monocytogenes* with a multiplicity of infection (MOI) of 10. The kinetics of luminescence were measured using the Synergy H1 multi-mode plate reader (Biotek). The area under the curve (AUC) was calculated with GraphPad Prism software V8.0.1 (San Diego, CA).

### Bacterial Burden Quantitation

WT and Trpm2^−/−^ mice were *i.v*. infected with 10^4^ CFU, animals were euthanized at 18 and 72 h post-infection (hpi), followed by the dissection of liver and spleen, organs were mashed and CFU quantified by serial dilution and plating on LB agar. The CFU were calculated and normalized to the weight of the organs. In some experiments, WT or Trpm2^−/−^ mice were injected *i.v*. with anti-Ly6G antibodies 1 day before bacterial infection and 2 days after bacterial infection for depletion of neutrophils.

### Cytokine Quantitation

For *in vivo* quantitation of inflammatory cytokines response, WT and Trpm2^−/−^ mice were *i.v*. infected with 10^4^ CFU of *L. monocytogenes*, blood samples were collected 18 and 72 hpi, Cytometric Bead Array (CBA) assay was performed to quantitate TNF-α, IL-6, IL-10, CCL2, IL-12, and IFN-γ according to the manufacturer's directions (BD), the samples were acquired with an LSR II flow cytometer (BD) and analyzed using the FCAP Array software V3.0 (BD).

For the *ex vivo* experiments, bone marrow from WT or Trpm2^−/−^ mice was collected and cultured for 5 days in presence of 10 nM of M-CSF (Biolegend) to induce macrophage (MΦ) differentiation. 10^6^ MΦ were then seeded and stimulated with 100 ng/ml of LPS or with *L. monocytogenes* (MOI of 5) overnight, the supernatants were collected and stored at −80°C. Bone marrow neutrophils were purified by negative selection, as described above, and 10^6^ neutrophils were seeded and stimulated with *L. monocytogenes* (MOI of 10) for 4 h, the supernatants were collected and stored at −80°C. Inflammatory cytokines (TNF-α, IL-1α, IL-1β, IL-6, IL-10) were quantitated in supernatants from cell cultures of MΦ and neutrophils by using the LEGENDplex mouse inflammation panel (Biolegend), the assay was performed according to the manufacturer's directions. The samples were acquired with an LSR II flow cytometer and analyzed using the LEGENDplex data analysis software (Biolegend).

### Immunofluorescence and Histopathology

WT, Trpm2^−/−^ or mice subjected to neutrophil depletion (anti-Ly6G 1 day before infection and 2 days post-infection) were infected with *L. monocytogenes*, 72 hpi livers and spleens were dissected, fixed, embedded in paraffin or optimal cutting temperature (OCT) compound and frozen in liquid nitrogen. Sections were cut (4 μm) and stained with rabbit anti-Listeria (Abcam) 1:100, rat anti-Ly6G (Biolegend) 1:100 or rat anti-Ly6C (Santa Cruz Biotechnology) 1:100 plus chicken anti-rabbit IgG Alexa Fluor 488 (Invitrogen) 1:500 and goat anti-rat IgG Alexa Fluor 594 (Invitrogen) 1:500. The slides were prepared with fluoroshield mounting medium with DAPI (Abcam). For the histopathological analysis, paraffin sections of spleens and livers were stained with Hematoxylin and Eosin (H&E) and analyzed by microscopy. The bacterial abscesses were counted in the median lobe of the livers at 72 hpi, whereas in the spleens the severity of the infection was evaluated by comparing the percentage of necrotic follicles.

### Flow Cytometry

WT or Trpm2^−/−^ mice were infected with 10^4^ CFU of *L. monocytogenes i.v*., liver or spleen were collected 18 and 72 hpi, the organs were disrupted and leukocytes were purified using Percoll 33% as previously described (23). Cells were stained with antibodies (dilution 1:100) (Biolegend) against CD45 Brilliant Violet 510, CD11b Brilliant Violet 785, Ly6G Brilliant Violet 605, Ly6C Brilliant Violet 421, F4/80 Brilliant Violet 711 and Live/Dead Blue (Invitrogen). Stained cells were acquired in an LSR II flow cytometer (BD) and analyzed with FlowJo V10.1 (Tree Star). The gating strategy for neutrophils was as follows: singlets>live cells>CD45+ >CD11+, Ly6G^high^ Ly6C^int^. The gating strategy for inflammatory macrophages was: singlets>live cells>CD45+ >CD11+, Ly6G- Ly6C^high^, F4/80-. Relative cell counts were assessed by using countBright absolute counting beads (Thermofisher).

### Degranulation Assay

WT and Trpm2^−/−^ bone marrow neutrophils were stimulated with 100 nM of PMA for 30 min, then fixed and stained with anti-mouse CD63 APC/Cy7 (Biolegend) 1:50. The cells were acquired by flow cytometry and analyzed with FlowJo software.

### Cell Death and NETosis Assay

WT or Trpm2^−/−^ bone marrow neutrophils were stimulated with 1 mM of H_2_O_2_ or *L. monocytogenes* (MOI of 10), dead cells were identified by staining with 1 μM of SyTOX green (impermeant to live cells). Neutrophils were acquired by flow cytometry and analyzed with FlowJo software. For the analysis of extracellular DNA in a plate, WT, Trpm2^−/−^ or Nox2^−/−^ bone marrow neutrophils were stimulated with 100 nM of PMA. Next, 1 μM of SyTOX green was added and the kinetic of extracellular DNA release was measured by a fluorescence plate reader (488/525 nm). The formation of Neutrophil Extracellular Traps (NETs) was evaluated by immunofluorescence. WT or Trpm2^−/−^ bone marrow neutrophils were seeded in coverslips and stimulated with 100 nM of PMA, 1 mM of H_2_O_2_ or *L. monocytogenes* (MOI of 10) for 3 h, cells were fixed and stained with rabbit anti-mouse Neutrophil Elastase (Abcam) 1:100, wheat germ agglutinin (WGA) Oregon 488 (Thermofisher) 1:1,000, Hoechst 33342 (Thermofisher) and goat anti-rabbit Alexa Fluor 594 (Abcam) 1:500. The slides were mounted with fluoroshield mounting medium (Abcam) and visualized by confocal microscopy.

### Intracellular Antimicrobial Killing Assay

10^6^ WT or Trpm2^−/−^ bone marrow neutrophils were infected with *L. monocytogenes* with a multiplicity of infection (MOI) of 10 for 45 min, cells were centrifuged at 335 × g for 5 min and the supernatant was removed. Infected neutrophils were seeded in 24 well plates and incubated for 3 h at 37°C. To quantify intracellular bacteria, neutrophils were lysed with 0.1% Triton X-100 (Sigma Aldrich, St. Louis, MO), and free bacteria were quantified by serial dilution on LB agar plates. Relative percent of antimicrobial killing was calculated dividing the inverse CFU obtained from WT neutrophils by CFU from Trpm2^−/−^ neutrophils and multiplied by 100.

### Measurement of Cytosolic Ca^2+^ in Neutrophils

For the analysis of intracellular Ca^2+^ mobilization by flow cytometry, bone marrow neutrophils were freshly isolated and stained with 10^−6^ M Fluo-4 AM (Invitrogen, Eugene, OR) for 45 min in the dark at RT, then cells were resuspended in Hank's Balanced Salt Solution (HBSS) buffer and aliquots were prepared with 5 × 10^5^ cells/tube, for some experiments, neutrophils were preincubated with 3 × 10^−3^ M EGTA. The kinetics of Ca^2+^ were recorded by collecting baseline levels, followed by the addition of 10^−7^ M N-formyl-methionyl-leucyl-phenylalanine (fMLP), (10^−3^, 5 × 10^−3^ or 10^−2^ M) H_2_O_2_, or *L. monocytogenes* (MOI of 10), the accumulation of intracellular free Ca^2+^ was assessed by FACS with an LSR II cytometer (BD, Franklin Lakes, NJ) for up to 300 s. The results were analyzed using FlowJo (Ashland, OR) and GraphPad Prism (San Diego, CA). For quantitative evaluation of Ca^2+^ responses, the areas under the curve (AUC) were calculated for each trace by using GraphPad Prism. For microscopic visualization of cytosolic Ca^2+^, bone marrow neutrophils were stained with 10^−6^ M Rhod-2 AM (Invitrogen, Eugene OR) for 45 min in the dark at RT, then cells were resuspended in HBSS buffer. Neutrophils were seeded in microscopy slides for 10 min at 37°C, the basal fluorescence was adjusted prior the stimulation with *L. monocytogenes* (MOI of 10), and the kinetics of intracellular Ca^2+^ were recorded up to 4 min by using a Zeiss LSM800 confocal microscope. Images and videos were analyzed using ImageJ (NIH, USA).

### Measurement of Membrane Potential

Bone marrow neutrophils were loaded with 10^−6^ M DiBAC_4_(3) (Thermofisher), for 30 min at 37°C, then cells were transferred to flow cytometer tubes in aliquots of 10^6^ neutrophils. The dye enters to depolarized cells, exhibiting an enhance in fluorescence. Conversely, hyperpolarization is indicated by a decrease in fluorescence.

The kinetics of membrane potential were started by the acquisition of basal levels (up to 60 s), followed by the addition of 10^−7^ M PMA or *L. monocytogenes* (MOI of 10), the curves were recorded up to 600 or 300 s, respectively, by using an LSR II cytometer (488, 505 LP 530/30 BP). The analysis was performed using FlowJo and GraphPad software. A quantitative analysis of membrane depolarization was obtained calculating the AUC by using GraphPad Prism software.

### qPCR

WT, Trpm2^−/−^ or mice treated with anti-Ly6G, were infected with *L. monocytogenes*, 72 h later, livers were collected, and a small portion was lysed with QIAzol (Qiagen). RNA was purified with columns (Qiagen) and cDNA was prepared with Super Script II reverse transcriptase (Thermofisher). For qPCR, 96 well plates were used with a customized Taqman design (Thermofisher, 4391528). Data were normalized using 18s RNA as a housekeeping gene and the fold changes were made by comparing non-infected with infected samples.

### Statistical Analysis

Data analysis were performed by using GraphPad Prism 8 (San Diego CA). Statistical evaluation was performed with ANOVA one way with Dunnett's, Tukey's or Sidak tests for comparison of multiple groups and Welch's *t*-test or multiple *t*-tests for comparing two data sets. A value of *p* < 0.05 was considered statistically significant.

## Results

### Trpm2^−/−^ Mice Are Susceptible to *L. monocytogenes* Infection

**Figure 1:**
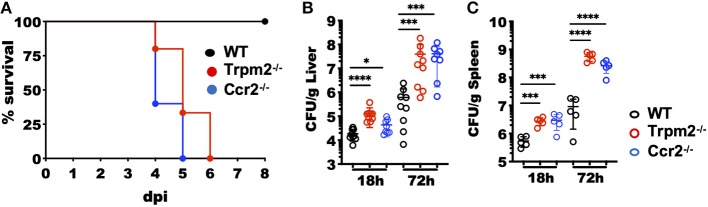
Increased susceptibility of Trpm2^−/−^ mice to *L. monocytogenes* infection. **(A)** Mice were *i.v*. infected with 10^4^ CFU of *L. monocytogenes* and survival monitored up to 8 days post-infection (dpi). Survival is represented by Kaplan–Meier survival curves for WT (*n* = 11), Ccr2^−/−^ (*n* = 5) and Trpm2^−/−^ (*n* = 15). Bacterial burden of mice infected with *L. monocytogenes* is shown at 18 and 72 hpi in **(B)** liver (WT and Trpm2^−/−^ *n* = 9, Ccr2^−/−^ *n* = 8) and **(C)** spleen (*n* = 5). Graphs show mean ± SD, statistical analysis was performed using ANOVA one way and Dunnett's multiple comparison test (*p < 0.05, ***p < 0.001, ****p < 0.0001).

The mouse model of systemic listeriosis has been used as a powerful tool to study innate and adaptive immune responses for decades (24). In the acute inflammatory phase, myeloid cells have a critical role in controlling the bacterial burden of *L. monocytogenes* infection (25, 26). To determine how TRPM2 channel modulates the innate inflammatory response induced against *L. monocytogenes* infection, we infected C57BL/6 (WT), Trpm2^−/−^ or Ccr2^−/−^ mice with a sublethal dose of *L. monocytogenes* (10^4^ CFU) and evaluated the susceptibility of Trpm2^−/−^ mice to infection. The Ccr2^−/−^ mice were used as a control, because the strain reported susceptibility to *L. monocytogenes* infection (27). As expected, WT mice were resistant to *L. monocytogenes* infection and 100% of the animals survived for the length of the experiment (8 dpi). In contrast, Ccr2^−/−^ mice were highly susceptible to *L. monocytogenes* infection and did not survive beyond 5 dpi, as reported previously (27). Similar to the Ccr2^−/−^ group, only 30% of the Trpm2^−/−^ mice survived 5 dpi and by 6 dpi 100% of this group had succumbed to the acute infection (Figure 1A). The Ccr2^−/−^ and Trpm2^−/−^ mice showed increased bacterial burden at 18 and 72 hpi in liver (Figure 1B) and spleen (Figure 1C) compared to WT mice. To analyze the time course of *L. monocytogenes* colonization and bacterial spreading in mice, we infected the animals using luminescently labeled *L. monocytogenes* Xen-32 and followed luminescence emission *in vivo* at 6 hpi. Trpm2^−/−^ mice showed greater levels of luminescence than WT mice when visualized in the ventral position (Supplementary Figure 1A), the relative luminescence was quantitated as shown (Supplementary Figure 1B). In addition, liver and spleen were dissected, and bacterial burden, was visualized in the organs *ex vivo* (Supplementary Figures 1C–E). Livers from Trpm2^−/−^ showed significantly higher bacterial burden, as compared to WT mice. Interestingly, spleens from WT mice showed greater concentration of *L. monocytogenes* spleens from Trpm2^−/−^ mice, suggesting bacterial containment in the spleen of the WT mice (Supplementary Figures 1C,E), as opposed to extensive bacterial spreading from the spleen to the liver in the Trpm2^−/−^ mice (Supplementary Figures 1C,E) at 18 and 72 hpi.

### TRPM2^−/−^ Phagocytes Differentially Migrate to the Site of Inflammation

**Figure 2:**
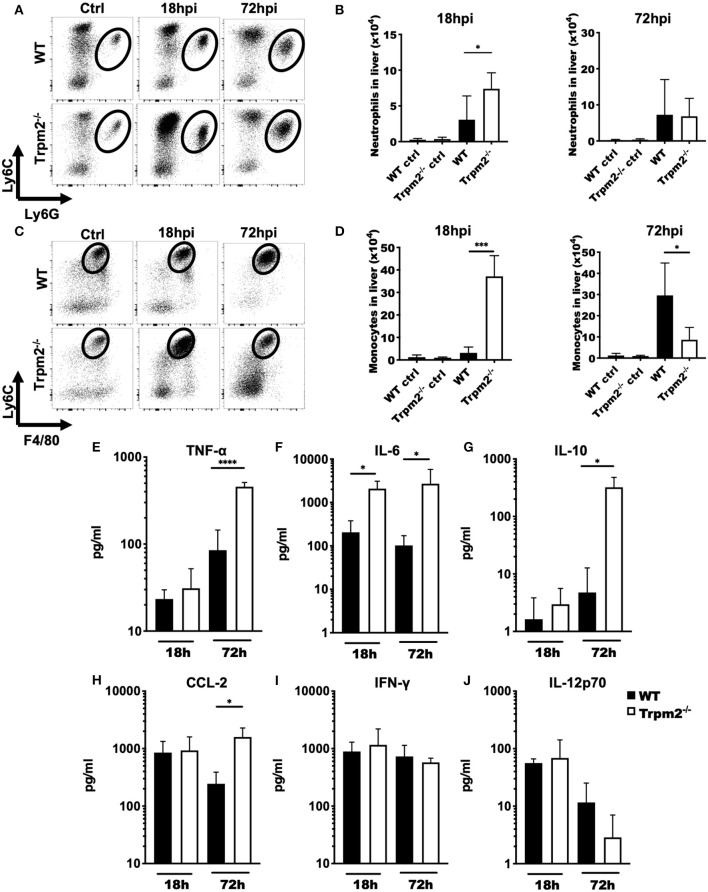
Trpm2^−/−^ inflammatory phagocytes migrate to infected organs and contribute to the development of systemic inflammation. WT and Trpm2^−/−^ mice were infected with *L. monocytogenes*. Livers were collected and disrupted, hepatocytes and erythrocytes were removed, and the remaining cells were immunostained. **(A)** Dot plots show the distribution of neutrophils (CD45^+^/CD11b^high^/Ly6G^high^/Ly6C^int^) in liver at 18 and 72 hpi in WT and Trpm2^−/−^ mice, **(B)** graphs show the total number of neutrophils in liver at 18 or 72 hpi (*n* = 5). **(C)** Distribution of monocytes in the liver at 18 and 72 hpi, **(D)** and the total number of monocytes in the liver (*n* = 5). Graphs show mean ± SD, the statistical analysis was performed using Welch's *t*-test (*p < 0.05, ***p < 0.001). **(E–J)** WT and Trpm2^−/−^ mice were infected with *L. monocytogenes* and blood was collected at 18 or 72 hpi, then serum was separated and TNF-α, IL-6, IL-10, CCL-2, IFN-γ, or IL-12p70 were quantified by flow cytometry (*n* = 5). Graphs show mean ± SD, the statistical analysis was performed using Welch's *t*-test (*p < 0.05, ****p < 0.001).

To determine the contribution of phagocytic cells to the susceptibility of Trpm2^−/−^ mice during *L. monocytogenes* infection, we analyzed the migration kinetics of myeloid cells to the site of infection. To this end, we collected the liver of WT and Trpm2^−/−^ infected mice at 18 and 72 hpi. Trpm2^−/−^ mice showed a greater number of neutrophils than WT at 18 hpi; however, the total number of neutrophils in the WT was only slightly larger at 72 hpi, but not significantly different than in Trpm2^−/−^ mice (Figures 2A,B). Trpm2^−/−^ mice also showed increased recruitment of inflammatory monocytes at 18 hpi as compared to WT, but WT mice reached a significantly larger number of inflammatory monocytes at 72 hpi (Figures 2C,D). Immunostainings of the liver from infected mice at 72 hpi showed an increased number of bacteria in the Trpm2^−/−^ organs, correlating with the larger amounts of recruited neutrophils (Supplementary Figure 1F). Similarly, increased recruitment of monocytes in the liver of Trpm2^−/−^ mice was observed (Supplementary Figure 1G), suggesting that myeloid cells may be participating in the exacerbated inflammatory responses of TRPM2 deficient mice upon *L. monocytogenes infection*.

### Trpm2^−/−^ Mice Infected With *L. monocytogenes* Develop Systemic Inflammation

Because Trpm2^−/−^ mice were highly susceptible to *L. monocytogenes* infection and succumbed as early as 4 dpi, we investigated whether these mice were undergoing septic shock. To achieve this goal, we analyzed the inflammatory cytokine profile of WT and Trpm2^−/−^ mice infected with *L. monocytogenes* at 18 and 72 hpi. Both, WT and Trpm2^−/−^ mice had no significant differences in blood levels of TNF-α at 18 hpi. However, blood levels of TNF-α were 5-fold increased in Trpm2^−/−^ mice, as compared to WT mice, at 72 hpi (Figure 2E). Trpm2^−/−^ mice also showed increased levels of IL-6 as early as 18 hpi (Figure 2F) and IL-6, IL-10 and CCL2 at 72hpi (Figures 2G,H), compared to WT mice. In contrast to a previous report (19), we did not find differences between WT and Trpm2^−/−^ mice in the blood levels of IFN-γ or IL-12 (Figures 2I,J). Augmented levels of IL-6 and TNF-α in Trpm2^−/−^ mice suggest a systemic inflammation in these animals, which likely results in a lethal septic shock during the course of *L. monocytogenes* infection.

### TRPM2 Ion Channel Regulates the Inflammatory Response in Neutrophils

**Figure 3:**
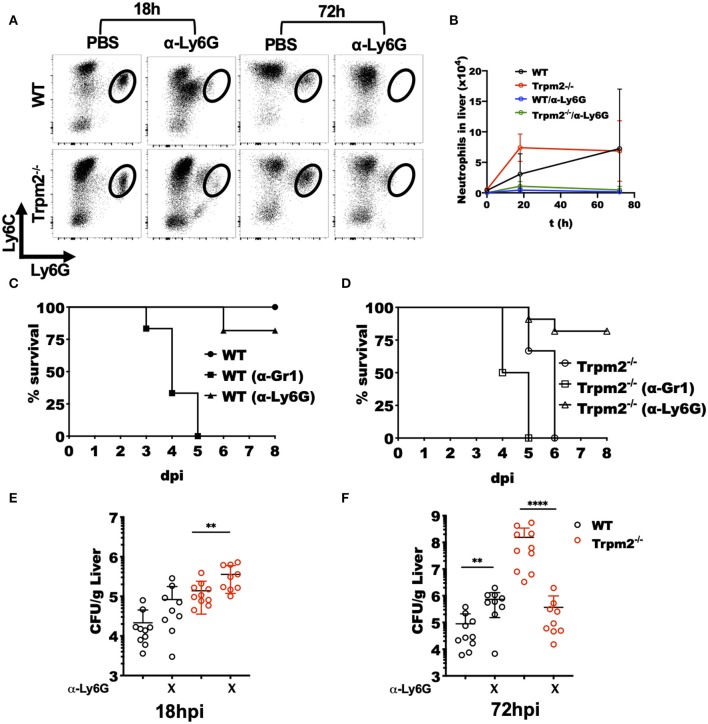
Depletion of Trpm2^−/−^ neutrophils promotes resistance to *L. monocytogenes* infection. Neutrophils (anti-Ly6G) were depleted in WT or Trpm2^−/−^ mice and infected with *L. monocytogenes*, the liver was collected at 18 or 72 hpi, and flow cytometry was performed to analyze inflammatory neutrophils. **(A)** The dot plots show the distribution of neutrophils in infected mice. **(B)** The graph shows kinetics of neutrophils total counts in liver for the groups of mice described in **(A)** (*n* = 5), mean ± SD. Survival analysis was performed on **(C)** WT or **(D)** Trpm2^−/−^ mice with neutrophils depletion (anti-Ly6G) or with neutrophils/monocytes depletion (anti-Gr1) up to day 8. WT (*n* = 6), Trpm2^−/−^ (*n* = 6), anti-Gr1 (*n* = 6 for WT and Trpm2^−/−^) and anti-Ly6G (*n* = 11 for WT and Trpm2^−/−^). The bacterial burden was quantified in liver at **(E)** 18 hpi or **(F)** 72 hpi (*n* = 9). Bar graphs show mean ± SD. The statistical analysis was performed using Welch's *t*-test (**p < 0.01, ****p < 0.0001).

To best define the specific contribution of phagocytes to the inflammatory response induced by *L. monocytogenes* in the Trpm2^−/−^ mice, we either depleted both neutrophils and monocytes or neutrophils only by treating the mice with antibodies anti-Gr-1 (RB6-8C5) or anti-Ly6G (1A8), respectively. Next, we infected the mice with *L. monocytogenes* and evaluated the susceptibility of depleted mice to the infection. To confirm the efficacy of cell depletion in the mice, we performed flow cytometry evaluation of cells from the liver of infected mice after cell depletion (Figures 3A,B). The treatment with anti-Ly6G effectively depleted the population of neutrophils in WT and Trpm2^−/−^ mice.

Depletion of neutrophils (anti-Ly6G), slightly increased susceptibility of WT mice to the bacterial infection (Figure 3C), a similar response to *L. monocytogenes* infection was previously described in neutrophil depleted mice (27). However, depletion of monocytes/neutrophils (anti-Gr1) rendered WT mice considerably more susceptible to *L. monocytogenes* infection (Figure 3C). Interestingly, neutrophils depletion in the Trpm2^−/−^ mice, increased their resistance to *L. monocytogenes* infection (Figure 3D), suggesting a predominant role for TRPM2^−/−^ neutrophils in orchestrating the inflammatory response elicited upon *L. monocytogenes* infection. The depletion of neutrophils/monocytes in Trpm2^−/−^ did not significantly change the susceptibility of the mice compared to non-depleted Trpm2^−/−^ mice, and those animals did not survive beyond day 5 after infection.

Examination of bacterial burden in livers from neutrophil depleted WT mice showed an increase in bacterial CFU at 18 hpi which reached the bacterial burden observed in Trpm2^−/−^ mice without any depletion. Neutrophils depletion in the Trpm2^−/−^ mice resulted in further increased bacterial burden in the liver compared to neutrophil depleted WT (Figure 3E). At 72 hpi, WT mice depleted for neutrophils had a slightly greater bacterial burden than the non-depleted WT mice. Interestingly, infected Trpm2^−/−^ mice treated with anti-Ly6G had a reduced bacterial burden at 72 hpi (Figure 3F), suggesting that the expression of TRPM2 ion channel in neutrophils impedes bacterial dissemination during the infection with *L. monocytogenes*.

**Figure 4:**
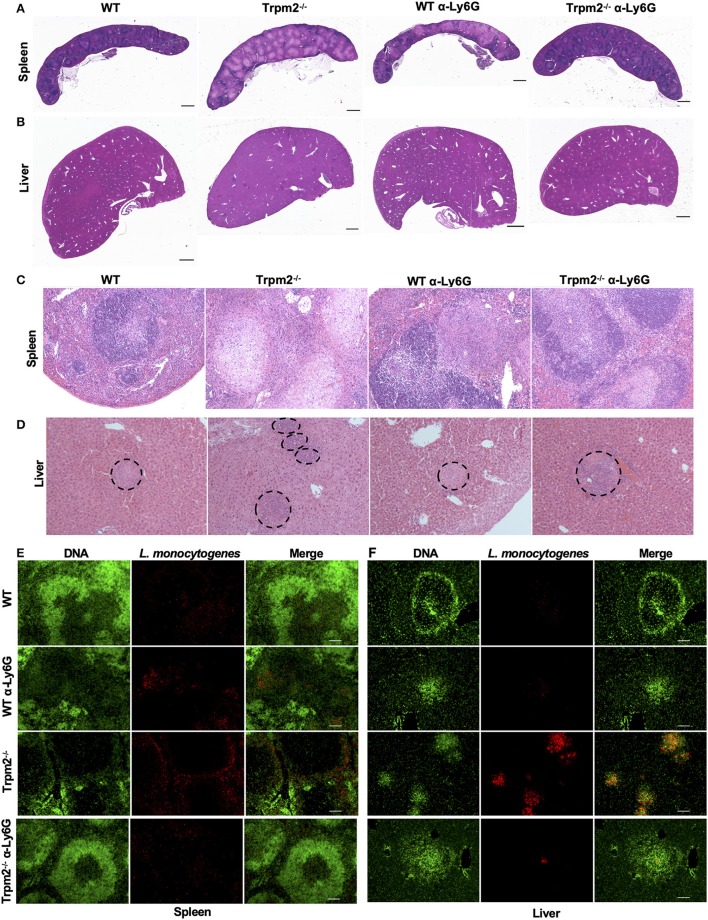
Depletion of neutrophils in Trpm2^−/−^ mice infected with *L. monocytogenes* reduces the bacterial burden and tissue damage. Neutrophils (anti-Ly6G) were depleted in WT or Trpm2^−/−^ mice, spleens and livers were dissected at 72 hpi, embedded in paraffin and stained with H&E. **(A)** Spleen and **(B)** liver of infected mice. Trpm2^−/−^ mice exhibited large areas of necrosis in the spleen and numerous abscesses in the liver compared to WT, depletion of neutrophils increased the areas of necrosis in spleen of WT mice while neutrophil depletion reduced the damage in Trpm2^−/−^ mice (scale bar indicates 1 mm). **(C)** The spleen of infected mice at 10X magnification, Trpm2^−/−^ mice showed a large area of necrosis in the B cell zone than other groups. **(D)** The livers of infected mice, dotted circles show the area of abscesses, depletion of neutrophils in Trpm2^−/−^ mice reduced the number of the abscesses. **(E)** Spleens or **(F)** livers were stained with DAPI (green) and anti*-L. monocytogenes* antibody (red) and visualized at 10X of magnification, Trpm2^−/−^ showed more positive staining areas for *L. monocytogenes* bacteria.

We next analyzed the immunopathology of spleens and livers from infected mice at 24 or 72 hpi by H&E staining. Low magnification images of spleens from infected Trpm2^−/−^ mice at 72 hpi, exhibited large areas of necrosis compared to WT mice (Figure 4A). Depletion of neutrophils in WT mice increased the areas of necrosis in the spleen. Interestingly, spleens from neutrophil-depleted Trpm2^−/−^ mice showed less necrotic areas in the spleen. Similar results were observed in the liver, where, Trpm2^−/−^ mice showed numerous abscesses (Figure 4B), however, the absence of neutrophils in Trpm2^−/−^ mice reduced the number of abscesses in the liver.

The microscopic images of infected spleens had minimal pathological changes at 18 hpi in both, WT and Trpm2^−/−^ mice (data not shown), however, Trpm2^−/−^ mice had large areas of caseous necrosis in the germinal center zones at 72 hpi (Figure 4C), such tissue damage was significantly smaller in infected WT mice and neutrophil-depleted Trpm2^−/−^ mice. Consistently, infected Trpm2^−/−^ mice displayed a greater percentage of spleen follicles with necrosis than WT mice. Interestingly, the depletion of neutrophils reduced the number of necrotic areas in the Trpm2^−/−^ mice, reaching similar levels as WT mice (Supplementary Figure 2A). Bacterial accumulation in the liver was observed in the abscess-like structures in the Trpm2^−/−^ mice (Figure 4D dotted circles). At 72 hpi Trpm2^−/−^ mice showed a large number of abscesses in the liver. In contrast, the livers from Trpm2^−/−^ mice that were depleted of neutrophils showed a significantly reduced number of abscesses compared to the Trpm2^−/−^ mice counterpart without neutrophils depletion (Figure 4D and Supplementary Figure 2B), suggesting that Trpm2^−/−^ neutrophils may facilitate bacterial dissemination and subsequent tissue damage in these animals.

To correlate bacterial tissue invasion with the observed tissue pathology, we used immunofluorescence to localize *L. monocytogenes* in the infected organs. The immunostaining revealed large spots of bacterial accumulation (red) in the spleens of Trpm2^−/−^ mice at 72 hpi, and the bacterial distribution was mainly localized in the white pulp (Figure 4E). Whereas, WT or neutrophil depleted WT mice showed reduced areas of bacterial accumulation, as compared to Trpm2^−/−^ mice, depletion of neutrophils in Trpm2^−/−^ mice resulted in even further reduction in bacterial dissemination in the spleen during the acute infection. Similar results were observed in livers of mice infected with *L. monocytogenes*, where Trpm2^−/−^ mice had a larger number of abscesses compared to WT mice (Figure 4F). Depletion of neutrophils in WT mice did not increase the dissemination of *L. monocytogenes* in the liver. However, depletion of neutrophils in Trpm2^−/−^ mice, drastically reduced the areas of infection in the liver, suggesting that deficiency of TRPM2 ion channel in neutrophils promotes bacterial dissemination in spleen and liver of mice infected with *L monocytogenes*.

### Depletion of Neutrophils Prevents Systemic Inflammation in Infected Trpm2^−/−^ Mice

**Figure 5:**
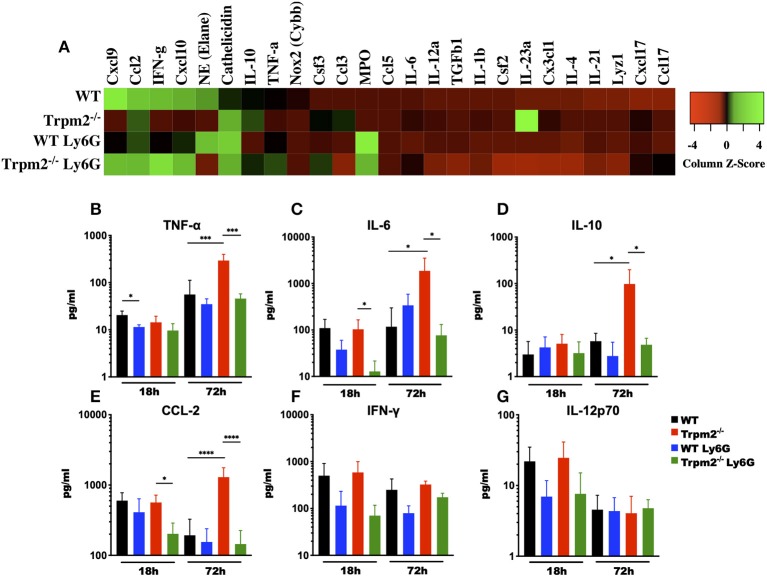
Depletion of neutrophils prevents the systemic inflammation in Trpm2^−/−^ mice infected with *L. monocytogenes*. **(A)** Gene expression analysis was performed on RNA collected from livers of WT (*n* = 3) and Trpm2^−/−^ (*n* = 3) mice, with or without depletion of neutrophils (WT *n* = 3, Trpm2^−/−^ *n* = 2), at 72 hpi. **(B–G)** Serum from infected mice was collected at 18 and 72 hpi, TNF-α, IL-6, IL-10, CCL-2, IFN-γ, or IL-12p70 were quantified by flow cytometry (*n* = 5). Bar graphs show mean ± SD. The statistical analysis was performed using ANOVA one way and Tukey's multiple comparison test (*p < 0.05, ***p < 0.001, ****p < 0.0001).

Because depletion of neutrophils in the Trpm2^−/−^ mice resulted in increased survival to *L. monocytogenes* infection, we sought to analyze the inflammatory microenvironment in the liver of these mice. We collected liver tissue from each group at 72 hpi and performed gene expression array analysis of selected inflammatory mediators. We found that Trpm2^−/−^ mice expressed increased levels of IL-23-α, Camp (Cathelicidin), IL-10, Csf3, and Ccl3, as compared to the other groups. Moreover, WT mice showed greater levels of Cxcl9, Elane (NE), IFN-γ, and Cxcl10, as compared to Trpm2^−/−^ mice (Figure 5A). Depletion of neutrophils in WT or Trpm2^−/−^ mice also modified the inflammatory liver microenvironment following infection. WT mice treated with anti-Ly6G showed increased NE, Cathelicidin, and MPO, but reduced Cxcl9 and IFN-γ. In addition, depletion of neutrophils in Trpm2^−/−^ mice increased the expression levels of Cxcl9, IFN-γ, and Cxcl10 but reduced the expression of IL-10 and IL-23-α compared to non-neutrophil depleted Trpm2^−/−^ mice (Figure 5A).

Due to infection lethality in some of our experimental groups, we hypothesized that those mice were undergoing septic shock. To test this, we analyzed the levels of various cytokines known to be involved in septic shock syndrome, including: TNF-α, IL-6, IL-10, CCL-2, IFN-γ, and IL-12 in the blood of mice infected with *L. monocytogenes*. Blood collected at 72 hpi from neutrophil depleted Trpm2^−/−^ mice contained reduced levels of TNF-α, as compared with non-neutrophil-depleted Trpm2^−/−^ mice (Figure 5B). The blood levels of TNF-α did not change significantly in neutrophil depleted WT mice, when compared to non-depleted WT group. Depletion of neutrophils in the WT mice initially yielded decreased levels in IL-6 at 18 hpi, but the levels of IL-6 were slightly higher in this group at 72 h (Figure 5C). Contrasting the results in the WT group, neutrophil depletion in Trpm2^−/−^ mice resulted in lower levels of IL-6 at 18 and 72 hpi (Figure 5C). Neutrophils depletion prevented the significant increase in IL-10 (Figure 5D) and CCL-2 (Figure 5E) blood levels seen with the Trpm2^−/−^ mice at 72 hpi. Interestingly, depletion of neutrophils in WT or Trpm2^−/−^ mice reduced slightly, but not significantly, the levels of IFN-γ at 18 and 72 hpi (Figure 5F). Similarly, the blood levels of IL-12 were only marginally reduced in both groups, neutrophil-depleted WT and neutrophil-depleted Trpm2^−/−^ mice at 18 hpi (Figure 5G), but no difference was observed at 72 hpi, as compared to non-depleted groups. These data suggest that the presence of neutrophils may be less impactful on the production of regulatory cytokines IFN-γ and IL-12, but TRPM2 function in neutrophils is critical to determine the course of inflammation during *L. monocytogenes* infection.

### TRPM2 Function Regulates Antimicrobial Responses in Neutrophils

**Figure 6:**
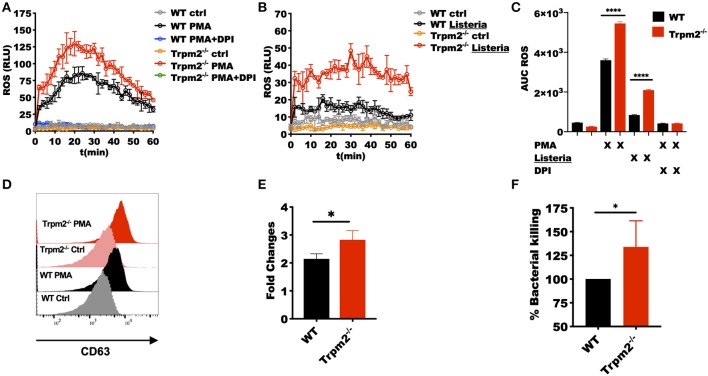
Trpm2^−/−^ phagocytes are hyperresponsive to *L. monocytogenes* infection. WT and Trpm2^−/−^ peritoneal neutrophils were stimulated with **(A)** 100 nM PMA or **(B)** *L. monocytogenes* at MOI of 10, some cells were pretreated with 10 μM of DPI. Oxidative burst was measured by adding 100 μM of luminol, chemiluminescence was measured on a plate reader (*n* = 3, mean ± SD). **(C)** The bar graphs show the area under the curve (AUC) of the ROS curves (*n* = 3, mean ± SD, ****p < 0.0001). **(D)** Bone marrow neutrophils were stimulated with 100 nM of PMA and primary granules release was analyzed by measuring the expression of CD63 on the cell surface 30 min after stimulation. **(E)** Shows the fold changes of WT and Trpm2^−/−^ granules release (*n* = 3, mean ± SD, *p < 0.05). **(F)** The intracellular antimicrobial killing was measured in WT and Trpm2^−/−^ neutrophils infected with *L. monocytogenes* (3 h), the graph shows the relative % killing of Trpm2^−/−^ neutrophils compared to WT (*n* = 8, mean ± SD, *p < 0.05). The statistical analysis was performed using Welch's *t*-test (*p < 0.05).

Our *in vivo* studies strongly suggest that neutrophils regulate the systemic inflammatory response, and therefore, determine the fate of the animals upon *L. monocytogenes* infection. We then questioned how the TRPM2 ion channel could be involved in the regulation of inflammation and whether such influence was due to alterations in the antimicrobial mechanisms of neutrophils. To address these questions, we purified peritoneal neutrophils and measured the neutrophils' oxidative response. We found that Trpm2^−/−^ neutrophils produced more oxidative products when cells were stimulated with phorbol 12-myristate 13-acetate (PMA) (Figure 6A) or *L. monocytogenes* compared to WT neutrophils (Figure 6B). The analysis of the area under the curve (AUC) of the ROS kinetics showed statistical significance between WT and Trpm2^−/−^ neutrophils (Figure 6C).

Next, we analyzed the mobilization of primary granules by measuring the expression of the primary granules marker CD63 (LAMP-3) in the cell membrane, and found that Trpm2^−/−^ neutrophils released more primary granules than WT after the cells were stimulated with PMA (Figures 6D,E), suggesting that the lack of TRPM2 induces changes in cytoskeleton and increased mobilization of intracellular vesicles in neutrophils. Due to the extensive tissue damage observed in Trpm2^−/−^ mice infected with *L. monocytogenes*, we sought to analyze the capabilities of Trpm2^−/−^ neutrophils to kill the bacteria. Surprisingly, we found that Trpm2^−/−^ neutrophils were more efficient at killing *L. monocytogenes* compared to WT neutrophils (Figure 6F).

### TRPM2 Channel Deficiency Results in Hyper Inflammatory Cytokine Response of Phagocytes

**Figure 7:**
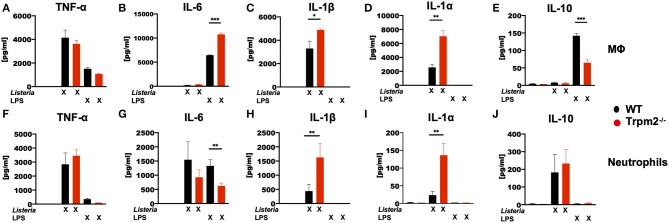
*L. monocytogenes* induces a hyperinflammatory cytokine profile in Trpm2^−/−^ phagocytes. WT or Trpm2^−/−^ bone marrow-derived macrophages (MΦ) were stimulated with 100 ng/ml of LPS or infected with *L. monocytogenes* (MOI of 5) overnight, the supernatants were collected and **(A)** TNF-α, **(B)** IL-6, **(C)** IL-1β, **(D)** IL-1α, and **(E)** IL-10 were quantified by flow cytometry (*n* = 3). WT or Trpm2^−/−^ bone marrow neutrophils were stimulated with 100 ng/ml of LPS or infected with *L. monocytogenes* (MOI of 10) for 4 h, the supernatants were collected and **(F)** TNF-α, **(G)** IL-6, **(H)** IL-1β, **(I)** IL-1α, and **(J)** IL-10 were quantified by flow cytometry (*n* = 4). Bar graphs represent the mean ± SD, the statistical analysis was performed by using Welch's *t*-test (*p < 0.05, **p < 0.01, ***p < 0.001).

To analyze whether the phagocytes were involved in the production of the inflammatory cytokines detected in mice infected with *L. monocytogenes*, bone marrow derived macrophages (MΦ) and neutrophils isolated from the bone marrow, were stimulated *in vitro* with LPS or with *L. monocytogenes*, and then the levels of TNF-α, IL-6, IL-1β, IL-1α, and IL-10 cytokines determined. Trpm2^−/−^ and WT MΦ showed similar levels of TNF-α under *L. monocytogenes* infection or LPS stimulation (Figure 7A). WT and Trpm2^−/−^ MΦ showed almost undetectable levels of IL-6 when cells were infected with *L. monocytogenes*, but Trpm2^−/−^ MΦ showed statistically significant greater levels of IL-6 under LPS stimulation, used as control (Figure 7B). In addition, *L. monocytogenes* induced significantly higher levels of IL-1β (Figure 7C) and IL-1α (Figure 7D) in Trpm2^−/−^ MΦ. Interestingly, the regulatory cytokine IL-10 was reduced in Trpm2^−/−^ MΦ infected with *L. monocytogenes* (Figure 7E), suggesting that Trpm2^−/−^ MΦ are more pro-inflammatory than WT MΦ. Furthermore, WT and Trpm2^−/−^ neutrophils showed similar levels of TNF-α (Figure 7F) and IL-6 (Figure 7G) under infection with *L. monocytogenes*. However, the cytokines derived from the inflammasome, IL-1β (Figure 7H) and IL-1α (Figure 7I) were elevated in Trpm2^−/−^ neutrophils. No differences were observed in the production of IL-10 (Figure 7J). Our results suggest a functional role for the TRPM2 cation channel in regulating the production of inflammatory cytokines in phagocytes. Thus, the expression of TRPM2 channel may prevent excessive local and systemic inflammatory responses mediated by immune phagocytes.

### TRPM2 Regulates Cell Death Pathways in Neutrophils

**Figure 8:**
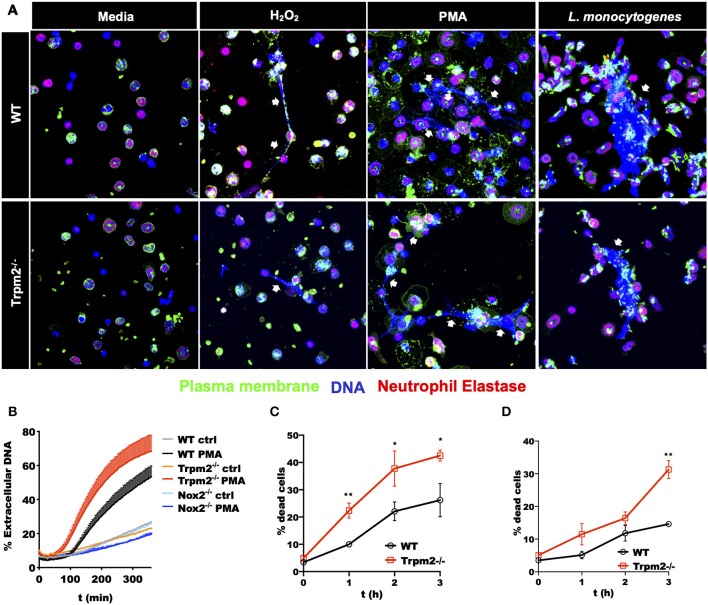
Stimulated Trpm2^−/−^ neutrophils undergo accelerated cell death. **(A)** The formation of NETs was induced by stimulating WT or Trpm2^−/−^ neutrophils with H_2_O_2_, PMA or *L. monocytogenes* for 3 h. The NETs structures (white arrows) were visualized by co-localized staining of Neutrophil Elastase and DNA (Hoechst 33342). The plasma membrane was stained using the lectin wheat germ agglutinin (WGA). Both WT and Trpm2^−/−^ neutrophils produced NETs under the stimulation with H_2_O_2_, PMA or *L. monocytogenes*. **(B)** WT, Trpm2^−/−^ or Nox2^−/−^ neutrophils were stimulated with PMA, SyTOX green (cell-impermeant dye) was added and kinetic of extracellular DNA (NETosis) was measured by fluorescence plate reader, the graph represents the mean ± SD. WT or Trpm2^−/−^ neutrophils were stimulated with **(C)** 1 mM of H_2_O_2_ or **(D)** *L. monocytogenes*, dead cells were identified by staining the cells in suspension with SyTOX green and analyzed by flow cytometry. The graphs show the % of dead cells in WT and Trpm2^−/−^ neutrophils after normalization with their respective unstimulated controls. The graphs show the mean ± SD (*n* = 3), the statistical analysis was performed using multiple *t*-tests (*p < 0.05, **p < 0.01).

The excesses of ROS products in Trpm2^−/−^ neutrophils suggested a potential reduction in the survival rate, and consequently, accelerated cell death of these cells. To evaluate the effect of increased ROS production in cell death activation pathways of Trpm2^−/−^ neutrophils, we determined the production of Neutrophils Extracellular Traps (NETs), which is linked to NETosis, a unique cell death pathway in neutrophils (28). NETosis in turn is associated to increased chronic inflammation (29, 30). To evaluate NET formation, we stimulated WT and Trpm2^−/−^ neutrophils with H_2_O_2_, a recognized activator of TRPM2, then detected extracellular DNA and neutrophil elastase by immunofluorescence. We observed fewer Trpm2^−/−^ neutrophils than WT producing NET fibers. Next, we stimulated the cells with PMA, or *L. monocytogenes* and found that Trpm2^−/−^ neutrophils were similarly capable of producing NET structures as compared to WT neutrophils (Figure 8A). In order to have a quantitative measurement of NETs, we analyzed the production of extracellular DNA by stimulating WT, Trpm2^−/−^ or Nox2^−/−^ neutrophils with PMA, dead cells and extracellular DNA were stained with SyTOX green (non-cell-permeable dye) and read by a fluorescence plate reader. We found that Trpm2^−/−^ neutrophils released more extracellular DNA than WT (Figure 8B). Because neutrophil NADPH oxidase can stimulate NETosis (31), neutrophils from Nox2^−/−^ mice were used as a negative control (32). As expected, NADPH deficient neutrophils did not produce NETs when stimulated with PMA (Figure 8B). Next, we evaluated the kinetics of cell death in cellular suspension by flow cytometry and confirmed that Trpm2^−/−^ neutrophils had increased cell death rate compared to WT, which peaked as early as 2 or 3 h when the cells were stimulated with either H_2_O_2_ (Figure 8C) or with *L. monocytogenes* (Figure 8D), respectively. Altogether, these findings suggest that TRPM2 channel functions as a negative regulator of antimicrobial inflammatory responses and NADPH-dependent cell death pathways in neutrophils.

### TRPM2 Regulates Ca^2+^ Signaling and Membrane Potential in Neutrophils

**Figure 9:**
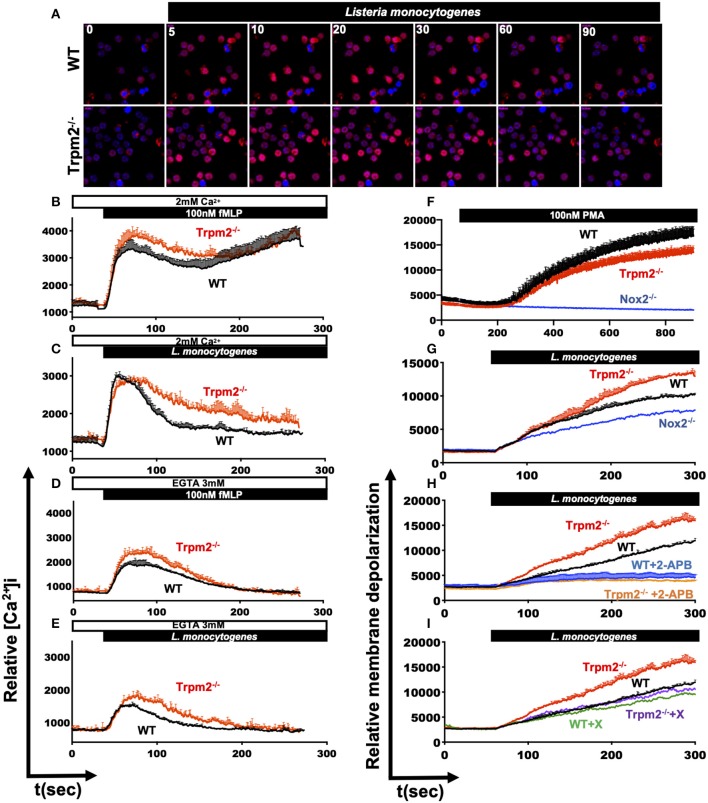
Dysregulated mobilization of Ca^2+^ in Trpm2^−/−^ neutrophils promotes increased membrane depolarization. WT or Trpm2^−/−^ neutrophils were stained with Rhod-2 AM and stimulated with *L. monocytogenes*, the kinetic was recorded by confocal microscopy, **(A)** shows the cytoplasmic levels of Ca^2+^ at 0, 5, 10, 20, 30, 60, and 90 s after stimulation. Neutrophils were stained with Fluo-4 AM and the experiments were recorded by flow cytometry, WT (black lines) or Trpm2^−/−^ (red lines) neutrophils were stimulated with **(B)** fMLP or **(C)** *L. monocytogenes* in media containing 2 mM of Ca^2+^. In some experiments, EGTA was added to the media to deplete free Ca^2+^, and the neutrophils were stimulated with **(D)** fMLP or **(E)** *L. monocytogenes*, Trpm2^−/−^ showed high levels of intracellular Ca^2+^ release. Graphs show mean ± SD (above), *n* = 3. For evaluation of membrane potential, WT and Trpm2^−/−^ neutrophils were stained with DiBAC_4_(3), the experiments were recorded by flow cytometry. WT and Trpm2^−/−^ neutrophils were stimulated with **(F)** PMA or **(G)** *L. monocytogenes*, Trpm2^−/−^ neutrophils showed higher levels of membrane depolarization than WT under *L. monocytogenes* stimulation. For some experiments, neutrophils were treated with **(H)** 2-APB or **(I)** Xestospongin C and stimulated with *L. monocytogenes*. Graphs show mean ± SD (above), *n* = 3.

The main known function of the TRPM2 ion channel is the modulation of Ca^2+^ entry, and consequently, it is expected that TRPM2 deficiency will impact major Ca^2+^ dependent cellular functions, including cell migration (33), oxidative stress (34, 35) and NADPH oxidase mediated cell death pathways (34). Our results indicate that TRPM2-deficient neutrophils exhibited increased inflammatory responses, which are mostly dependent on Ca^2+^ signaling (8). Therefore, we sought to determine the contribution of TRPM2 to Ca^2+^ entry triggered by PAMPs recognition in neutrophils. First, we stained neutrophils with Rhod-2 AM and analyzed the kinetic of intracellular Ca^2+^ mobilization by confocal microscopy. We observed that both, WT and Trpm2^−/−^ neutrophils were able to rapidly increase the cytoplasmic concentration of Ca^2+^ ([Ca^2+^]i) when the cells were stimulated with *L. monocytogenes* (Figure 9A). To best resolve the kinetics of Ca^2+^ mobilization during the time course of infection, we next stained the neutrophils with Fluo-4 AM and continuously recorded the events for 5 min by flow cytometry. Unexpectedly, Trpm2^−/−^ neutrophils stimulated with fMLP, responded with greater levels of [Ca^2+^]i than WT neutrophils (Figure 9B). Similar results were obtained when neutrophils were stimulated with *L. monocytogenes* (Figure 9C). When Ca^2+^ was depleted from the media by the addition of EGTA, Trpm2^−/−^ neutrophils showed slightly higher intracellular Ca^2+^ release than WT neutrophils under stimulation with fMLP (Figure 9D) or *L. monocytogenes* (Figure 9E). In neutrophils stimulated with H_2_O_2_ the concentration of 1mM, was insufficient to induce Ca^2+^ entry in neutrophils (Supplementary Figure 3A). However, Trpm2^−/−^ neutrophils showed a reduction of [Ca^2+^]i when the cells were stimulated with 5 mM of H_2_O_2_ (Supplementary Figure 3B) or with 10 mM of H_2_O_2_ (Supplementary Figure 3C), as compared to WT. The calculation of the area under the curve (AUC) showed statistical significance between WT and Trpm2^−/−^ neutrophils stimulated with fMLP (Supplementary Figure 3D), *L. monocytogenes* (Supplementary Figure 3E) or H_2_O_2_ (Supplementary Figure 3F). These findings suggest that the absence of TRPM2 ion channel in neutrophils modifies the regulation of PAMPs-dependent mobilization of Ca^2+^, possibly due to the engagement of alternative Ca^2+^ entry channels as a compensatory mechanism to maintain the Ca^2+^ homeostasis in neutrophils.

Because changes in the cellular membrane potential is a key physiological response in immune cells, which is tightly linked to in and out ion mobilization, and during the induction of oxidative stress (36). We evaluated the impact of TRPM2 function on the relative membrane potential of neutrophils directly in response to *L. monocytogenes* infection *in vitro*. To achieve this goal, we loaded the cells with the slow-response potential-sensitive probe DiBAC_4_(3), which responds to increases in cell depolarization by an increment in fluorescence (36). We recorded the fluorescence kinetics by flow cytometry and found that when stimulated with PMA, WT neutrophils had increased membrane depolarization, as compared to Trpm2^−/−^ neutrophils (Figure 9F). The analysis of the AUC indicated statistical significant difference between WT and Trpm2^−/−^ neutrophils stimulated with PMA (Supplementary Figure 3G). Trpm2^−/−^ neutrophils however, showed significant increases in membrane depolarization relative to WT cells, when stimulated with *L. monocytogenes* (Figure 9G and Supplementary Figure 3H). In contrast, Nox2^−/−^ neutrophils, which lack NADPH oxidase function, did not considerably increase membrane depolarization (Figure 9G). These results suggest that *L. monocytogenes* induces extensive membrane depolarization of neutrophils, and that TRPM2 regulates membrane depolarization in neutrophils, partially dependent on the NADPH oxidase complex induced by *L. monocytogenes*. To further analyze the impact of Ca^2+^ mobilization on membrane potential, we pretreated WT or Trpm2^−/−^ neutrophils with 2-APB, a wide spectrum inhibitor of TRP channels, and then stimulated the cells with *L. monocytogenes* (Figure 9H). Inhibition of generic TRP channels prevented membrane depolarization of both, WT and Trpm2^−/−^ neutrophils (Supplementary Figure 3I).

Moreover, pretreatment with Xestospongin C, an inhibitor of the IP_3_-dependent Ca^2+^ release pathway, slightly reduced membrane depolarization in WT neutrophils activated with *L. monocytogenes* (Figure 8I). Interestingly, Trpm2^−/−^ neutrophils treated with Xestospongin C reduced membrane depolarization levels similar to that of WT neutrophils, suggesting that in the absence of TRPM2 channel activity over-activation of the IP_3_-dependent Ca^2+^ release pathway, and subsequent store-operated Ca^2+^ entry may occur. Overall, our data suggest that the absence of TRPM2 channels in neutrophils causes an increase in membrane depolarization and Ca^2+^ overload, which favors the cascade of hyperinflammatory signals observed in the Trpm2^−/−^ neutrophils.

## Discussion

The activation of TRPM2 cation channel has emerged as an important cell-mechanism that regulates inflammation in phagocytes (13, 14, 16, 21, 37, 38). Because TRPM2 has been associated with oxidative responses, it becomes critical to understand the functional role of this channel in modulating antimicrobial effector mechanisms of phagocytic cells. Here, we focused on defining the specific contribution of the TRPM2 channel to the antimicrobial and inflammatory function of neutrophils in response to *L. monocytogenes* infection.

Previously, Knowles et al. reported that Trpm2^−/−^ mice were more susceptible than WT mice to *L. monocytogenes* infection (19). This report suggested that increased susceptibility was due to reduced production of IFN-γ, but when the mice received recombinant IFN-γ prior to infection, the Trpm2^−/−^ mice recovered resistance to *L. monocytogenes* infection (19). However, the study did not address how phagocytes participate in the pathobiology of the infection or the induction of systemic inflammation by *L. monocytogenes* infection in Trpm2^−/−^ mice. Unlike the Knowles et al. work, we focused our studies on the acute phase of the infection in an effort to understand the cellular events leading the mice to develop a systemic failure. We confirmed that indeed Trpm2^−/−^ mice were more susceptible than WT mice to *L. monocytogenes* infection. Since Trpm2^−/−^ mice developed systemic inflammation and succumbed at the acute phase of the infection, we first investigated how phagocytic cells contribute to the pathobiology leading to lethality in these mice. Furthermore, investigating the migration dynamics of phagocytic cells upon *L. monocytogenes* infection, we observed increased neutrophil and monocyte migration during the first 24 hpi in the liver of Trpm2^−/−^ mice, as compared to WT mice. Those inflammatory cells persisted in the liver of Trpm2^−/−^ mice longer than in WT mice, along with the deteriorating symptoms in those animals.

In addition to the cellular response, it is known that some inflammatory cytokines are critical for controlling the infection with *L. monocytogenes*, including IFN-γ (39), IL-12 (40), TNF-α (41) IL-6 (42), however, high levels of those inflammatory mediators can result in a lethal cytokine storm (43). In this study, the systemic inflammatory response developed by Trpm2^−/−^ mice was characterized by elevated levels of TNF-α, IL-6, IL-10, CCL-2 in blood, but surprisingly, no differences were observed in IFN-γ or IL-12 within the first 72 h post-infection. Nonetheless, better prognosis markers during bacterial infections may be IL-10 and IL-6 (44). Increased blood levels of IL-6 and IL-10 have been clinically related to the high rate of mortality in patients with severe sepsis (44–46). Also, systemic levels of IL-10 appear to facilitate bacterial persistence and dissemination within the host during infections caused by intracellular bacteria or by pathogens that modulate the inflammatory responses (47, 48). Indeed, increased levels of IL-10 have been linked to the progression of *L. monocytogenes* infections (48). It is, therefore, possible that increased production of IL-10 in the Trpm2^−/−^ mice, rather than a deficiency in IFN-γ or IL-12, may be associated to the susceptibility of the Trpm2^−/−^ mice upon *L. monocytogenes* infection.

Previously, neutrophils had been considered not to be essential in the natural resistance against *L. monocytogenes* infection (27), however, another group demonstrated the importance of neutrophils during the primary and secondary responses against *L. monocytogenes* (25). Our results are in good agreement with those later findings. Initially, we did not see increased susceptibility in WT mice after neutrophil depletion, however, neutrophil-depleted Trpm2^−/−^ mice developed resistance to *L. monocytogenes* infection. The extended survival of neutrophil-depleted Trpm2^−/−^ mice was also accompanied by reduced levels of bacterial burden in the liver and the spleen. Similarly, the levels of systemic inflammatory cytokines were reduced in Trpm2^−/−^ mice without neutrophils, suggesting that TRPM2^−/−^ neutrophils promote a state of hyper-inflammation, possibly related directly to ion homeostasis imbalance in these cells, which might contribute to the dissemination of *L. monocytogenes*.

Similar exacerbated inflammatory response was already observed in the Trpm2^−/−^ mice by our group, in a model of gastric infection by *H. pylori* infection (14), and by other investigators using distinct models of infection including, lung infection induced by *P. aeruginosa* (38), sepsis-induced by *E. coli* (49) or polymicrobial sepsis (50). Moreover, non-infectious models of inflammation have also added experimental evidence demonstrating that TRPM2 deficient animals cannot efficiently control inflammatory responses. For example, Trpm2^−/−^ mice succumbed to LPS challenge in a lung inflammatory model (20), Also, skin inflammation is aggravated in Trpm2^−/−^ mice in a model induced by LPS and TNF-α (16). In all these referenced studies, the increased inflammation was a consistent feature observed in the absence of TRPM2 ion channel, further supporting the paradigm of an anti-inflammatory functional role of TRPM2 (20, 51), as opposed to the paradigm favoring a pro-inflammatory function for TRPM2 in phagocytes (21, 51).

The infection with *L. monocytogenes* in Trpm2^−/−^ mice was characterized by neutrophilia, bacterial dissemination and acute tissue pathology in the liver and spleen of these mice, therefore, we sought to determine how the TRPM2 cation channel might regulate the antimicrobial response and inflammation in neutrophils. Our initial findings revealed that Trpm2^−/−^ neutrophils had increased effector functions, which included augmented production of ROS, enhanced released of primary granules, and likely, increased production of NETs than WT neutrophils, in response to *L. monocytogenes* infection. Altogether, these data suggested a direct functional role for the TRPM2 ion channel in the regulation of neutrophil's antimicrobial and inflammatory pathways. Despite the increased susceptibility of Trpm2^−/−^ mice to *L. monocytogenes* infection, TRPM2^−/−^ neutrophils exhibited increased capacity to kill these bacteria *in vitro*, which suggested that uncontrolled inflammation, rather than deficient bacterial killing, is responsible for exacerbated pathology resulting in the death of the Trpm2^−/−^ mice. Our previous findings showed that Trpm2^−/−^ macrophages had a similar marked inflammatory profile, which was associated with increased chronic gastric inflammation induced by *H. pylori* infection in Trpm2^−/−^ mice (14). It is, therefore, possible that in addition to neutrophils, macrophages may also contribute to the hyper inflammation observed in the tissue microenvironment upon bacterial infection in Trpm2^−/−^ mice. Notably, neutrophils also showed increased levels of cytosolic Ca^2+^ upon bacterial stimulation, in agreement with our published findings in TRPM2 deficient macrophages (14).

Stimulated neutrophils activate the membrane-associated NADPH oxidase (NOX2) resulting in a powerful oxidative burst, which constitute the central host defense mechanism in neutrophils (36, 52). We and others have proposed that activation of TRPM2 cation channel function downregulates NADPH oxidase activity, via a mechanism linked to membrane depolarization in phagocytes. Thus, activation of TRPM2 results in dampening of the NADPH oxidase-mediated ROS production through depolarization of the plasma membrane in WT phagocytes (20), whereas PMA or bacterial stimulation of Trpm2^−/−^ macrophages yielded increased levels of ROS (14). Therefore, as expected, the absence of TRPM2 channel in the Trpm2^−/−^ neutrophils also resulted in elevated NADPH oxidase activity and abundant ROS production, which correlated with the increase in membrane depolarization upon *L. monocytogenes* stimulation, as we show in Figures 9F–I, and likely the augmented NETosis observed in Figure 8, in response to PMA stimulation. Furthermore, the excessive membrane depolarization was prevented when 2-APB was added to Trpm2^−/−^ neutrophils, suggesting that in the absence of TRPM2 ion channels, additional plasma membrane channels, including members of the TRP family (10–12) or store-operated Ca^2+^ channels (7, 9), can be activated, and likely compensate for the entry of Ca^2+^ in these cells. The activation of plasma membrane Ca^2+^ channels will contribute to Ca^2+^ overloading and the overall hyperresponsiveness of Trpm2^−/−^ neutrophils. Consequently, the lack of TRPM2 channel-mediated function will result in the increased inflammation observed in Trpm2^−/−^ mice under *L. monocytogenes* infection.

Hence, TRPM2-mediated calcium influx in neutrophils is an essential mechanism for the regulation of the antimicrobial and inflammatory effector functions of these cells, including oxidative stress responses. The role of TRPM2 as an oxidant sensor has been extensively demonstrated in multiple cell types, including cancer cells [reviewed in (51)], where inhibition of the channel causes dysfunctional cellular bioenergetics, increased production of ROS, and impaired DNA repair leading to increased cell death (53, 54). It is therefore possible that, in addition to modulating Ca^2+^ mobilization, membrane depolarization, and NADPH activity, TRPM2 also controls neutrophils' oxidative burst, and oxidant dependent cell death, by scavenging the excess of harmful oxidants, such as H_2_O_2_ produced in response to bacterial stimulation. Altogether, the multiple key functions of TRPM2 appear to define the dynamic effector response of neutrophils during the onset of infection and/or inflammatory processes.

In summary, we propose that the TRPM2 ion channel functions as a global modulator of inflammation in neutrophils, by reducing the oxidative response and regulating Ca^2+^ influx and membrane depolarization in these cells. Thereby, TRPM2 could be a target aiming to modulate the pathology of inflammatory diseases were neutrophils are critical mediators of inflammation and aggravated tissue damage.

## Data Availability Statement

The raw data supporting the conclusions of this article will be made available by the authors, without undue reservation, to any qualified researcher.

## Ethics Statement

The Institutional Animal Care and Use Committee (IACUC) at the Abigail Wexner Research Institute of Nationwide Children's Hospital approved all animal experiments to ensure the humane care and ethical use of animals (IACUC protocol # 00505AR). All mice studies were performed in strict accordance with the National Institutes of Health standards as set forth in the Guide for the Care and Use of Laboratory Animals [DHSS Publication No. (NIH) 85–23].

## Author Contributions

FR-A and SP-S conceived the study, designed the experiments, and wrote the manuscript. FR-A, JR-R, and KB performed the experiments. FR-A analyzed the data and prepared the figures. FR-A, JR-R, KB, and SP-S edited the manuscript. SP-S provided financial resources and supervised the investigation.

### Conflict of Interest

The authors declare that the research was conducted in the absence of any commercial or financial relationships that could be construed as a potential conflict of interest.

## Supplementary Material

Supplementary Figure 1 Faster dissemination of *L. monocytogenes* in Trpm2^−/−^ mice induces an acute myeloid inflammatory response in the liver. **(A)** WT and Trpm2^−/−^ mice were infected with *L. monocytogenes* Xen-32 and luminescence was visualized in ventral position at 6 hpi, **(B)** luminescence was quantified as photons/s (*n* = 9). **(C)** Liver (*n* = 10) and spleen (*n* = 10) were dissected and visualized, graphs show the photons/s of **(D)** liver and **(E)** spleen. Frozen sections of livers from mice infected with *L. monocytogenes* at 72 hpi were stained with anti-*L. monocytogenes*, anti-Ly6G or anti-Ly6C, **(F)** shows the interaction of neutrophils with *L. monocytogenes* in the liver and **(G)** the interaction between monocytes and *L. monocytogenes*. Images were acquired at 10X (^*^*p* < 0.05, ^**^*p* < 0.01).

Supplementary Figure 2 Depletion of neutrophils in Trpm2^−/−^ mice results in reduced tissue pathology. Neutrophils (anti-Ly6G) were depleted in WT or Trpm2^−/−^ mice 1 day prior to infection and 2 days after infection, spleens and livers were dissected at 72 hpi, embedded in paraffin and stained with H&E. **(A)** The graph shows the percentage of spleen follicles with necrosis at 72 hpi (*n* = 3). The percentage of follicles with necrosis in the spleen was significantly larger in Trpm2^−/−^ mice compared to WT, the depletion of neutrophils however, reduced the percentage of follicles with necrosis in the spleen of Trpm2^−/−^ mice. **(B)** The quantitation of abscesses/median lobe in livers of mice 72 hpi (*n* = 3) showed a significantly increased number of abscesses in the liver of Trpm2^−/−^ mice compared to WT, but the depletion of neutrophils drastically reduced the number of abscesses in livers from Trpm2^−/−^ mice **(B)**. The bar graphs show mean ± SD, the statistical analysis was performed using one-way ANOVA and Tukey's multiple comparison tests (^*^*p* < 0.05).

Supplementary Figure 3 *L. monocytogenes* induces increased cytosolic levels of Ca^2+^ and membrane depolarization in Trpm2^−/−^ neutrophils. Bone marrow neutrophils were stained with Fluo-4 AM, aliquoted in HBSS containing Ca^2+^ and Mg^2+^ and stimulated with **(A)** 10 mM H_2_O_2_, **(B)** 5 mM H_2_O_2_ or **(C)** 1 mM H_2_O_2_. The kinetics of intracellular Ca^2+^ levels were recorded by flow cytometry up to 300 s (*n* = 3), the kinetics are shown as mean ± SD (upper). The areas under the curve (AUC) of kinetics of intracellular Ca^2+^ were quantified (the kinetics are shown in the Figure 9), The bar graphs show the AUC of neutrophils stimulated with **(D)** fMLP or **(E)** *L. monocytogenes*, in media containing Ca^2+^ or when Ca^2+^ was depleted by the addition of EGTA (*n* = 3). The **(F)** shows the AUC of the kinetics of intracellular Ca^2+^ when neutrophils were stimulated with 10, 5, or 1 mM of H_2_O_2_ (*n* = 3), the graphs show the mean ± SD, the statistical analysis was performed with Welch's *t*-test (^**^*p* < 0.01, ^***^*p* < 0.001). Bone marrow neutrophils were stained with Dibac_4_(3) in order to analyze membrane depolarization by flow cytometry. The kinetics of membrane depolarization are shown in Figures 9F–I. The bar graphs show the AUC of the kinetics of membrane depolarization when neutrophils were stimulated with **(G)** PMA, **(H)** *L. monocytogenes*, or **(I)** *L. monocytogenes* plus 2-APB or Xestospongin C. The graphs show the mean ± SD (*n* = 3). The statistical analysis was performed by Welch's *t*-test **(G,H)** or with ANOVA one-way and Sidak multiple comparisons (^****^*p* < 0.0001).
