# Supplementary figures and images for: The TRPM2 Ion Channel Regulates Inflammatory Functions of Neutrophils During Listeria monocytogenes Infection
Source: Front Immunol. 2020 Feb 4;11:97. doi: 10.3389/fimmu.2020.00097 (PMC7010865; doi:10.3389/fimmu.2020.00097)

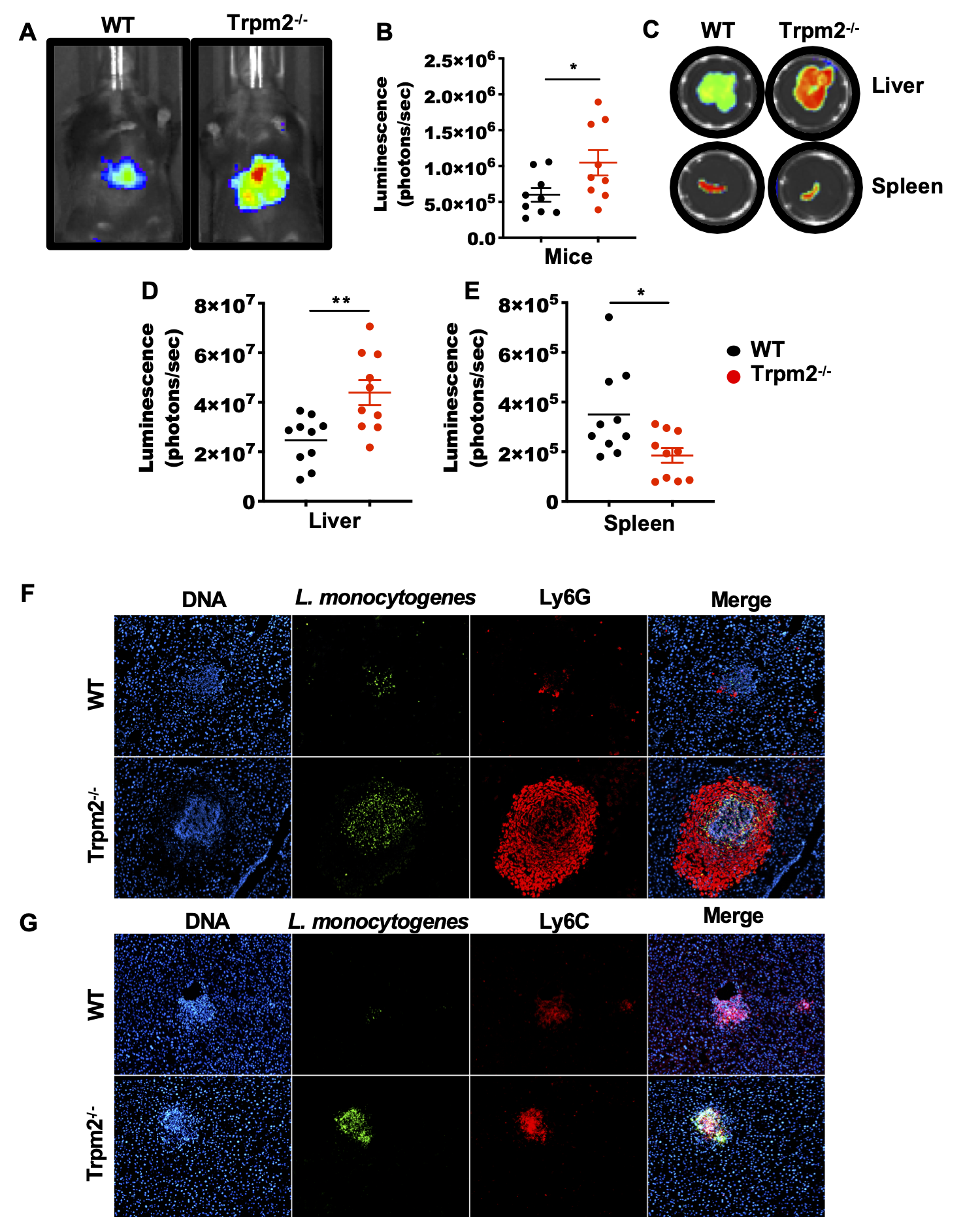

Supplement: Supplementary Figure 1 — Faster dissemination of L. monocytogenes in Trpm2−/− mice induces an acute myeloid inflammatory response in the liver. (A) WT and Trpm2−/− mice were infected with L. monocytogenes Xen-32 and luminescence was visualized in ventral position at 6 hpi, (B) luminescence was quantified as photons/s (n = 9). (C) Liver (n = 10) and spleen (n = 10) were dissected and visualized, graphs show the photons/s of (D) liver and (E) spleen. Frozen sections of livers from mice infected with L. monocytogenes at 72 hpi were stained with anti-L. monocytogenes, anti-Ly6G or anti-Ly6C, (F) shows the interaction of neutrophils with L. monocytogenes in the liver and (G) the interaction between monocytes and L. monocytogenes. Images were acquired at 10X (*p < 0.05, **p < 0.01). [file Image_1.TIFF]

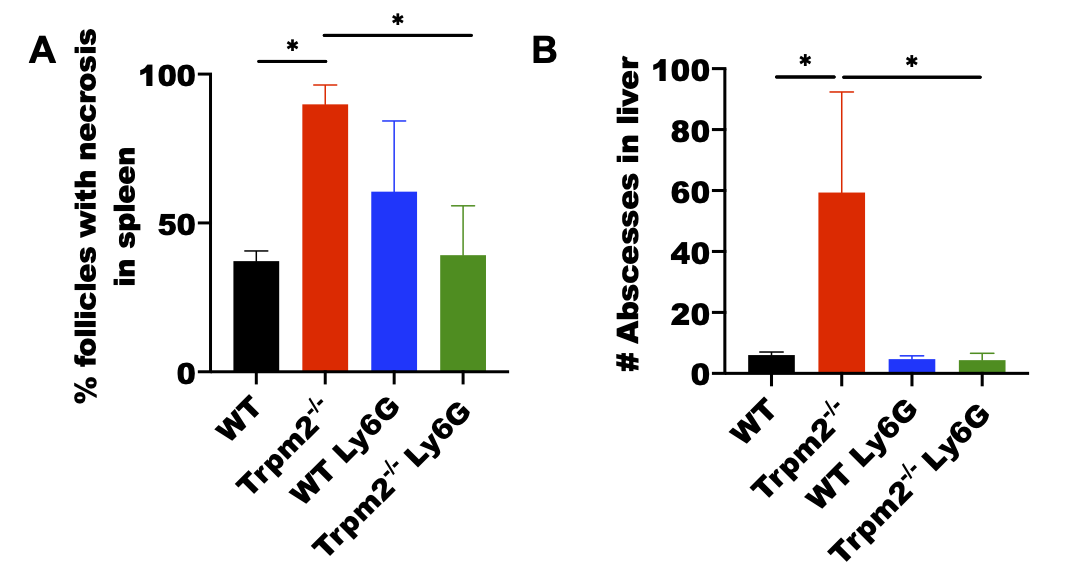

Supplement: Supplementary Figure 2 — Depletion of neutrophils in Trpm2−/− mice results in reduced tissue pathology. Neutrophils (anti-Ly6G) were depleted in WT or Trpm2−/− mice 1 day prior to infection and 2 days after infection, spleens and livers were dissected at 72 hpi, embedded in paraffin and stained with H&E. (A) The graph shows the percentage of spleen follicles with necrosis at 72 hpi (n = 3). The percentage of follicles with necrosis in the spleen was significantly larger in Trpm2−/− mice compared to WT, the depletion of neutrophils however, reduced the percentage of follicles with necrosis in the spleen of Trpm2−/− mice. (B) The quantitation of abscesses/median lobe in livers of mice 72 hpi (n = 3) showed a significantly increased number of abscesses in the liver of Trpm2−/− mice compared to WT, but the depletion of neutrophils drastically reduced the number of abscesses in livers from Trpm2−/− mice (B). The bar graphs show mean ± SD, the statistical analysis was performed using one-way ANOVA and Tukey's multiple comparison tests (*p < 0.05). [file Image_2.TIFF]

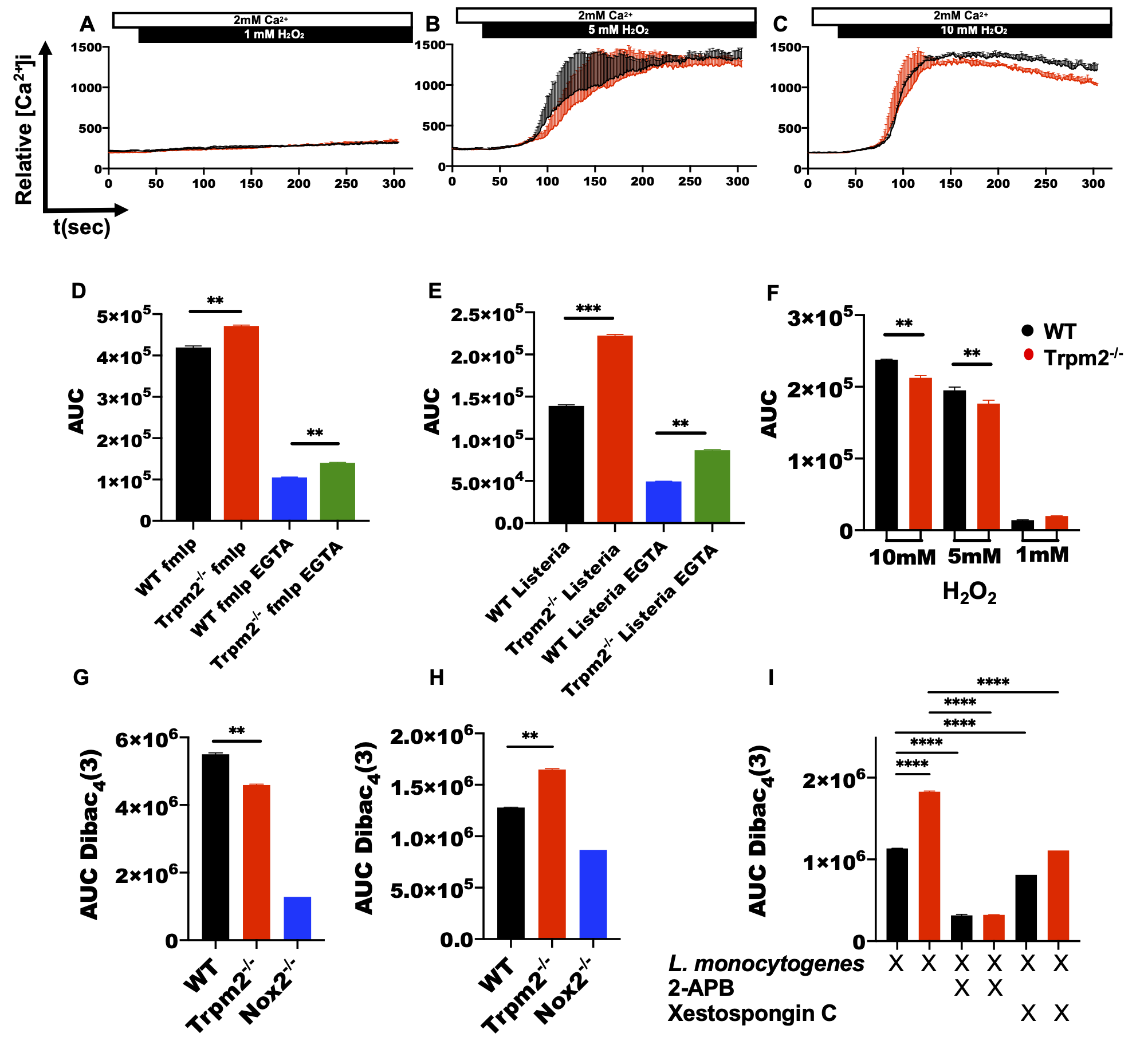

Supplement: Supplementary Figure 3 — L. monocytogenes induces increased cytosolic levels of Ca2+ and membrane depolarization in Trpm2−/− neutrophils. Bone marrow neutrophils were stained with Fluo-4 AM, aliquoted in HBSS containing Ca2+ and Mg2+ and stimulated with (A) 10 mM H2O2, (B) 5 mM H2O2 or (C) 1 mM H2O2. The kinetics of intracellular Ca2+ levels were recorded by flow cytometry up to 300 s (n = 3), the kinetics are shown as mean ± SD (upper). The areas under the curve (AUC) of kinetics of intracellular Ca2+ were quantified (the kinetics are shown in the Figure 9), The bar graphs show the AUC of neutrophils stimulated with (D) fMLP or (E) L. monocytogenes, in media containing Ca2+ or when Ca2+ was depleted by the addition of EGTA (n = 3). The (F) shows the AUC of the kinetics of intracellular Ca2+ when neutrophils were stimulated with 10, 5, or 1 mM of H2O2 (n = 3), the graphs show the mean ± SD, the statistical analysis was performed with Welch's t-test (**p < 0.01, ***p < 0.001). Bone marrow neutrophils were stained with Dibac4(3) in order to analyze membrane depolarization by flow cytometry. The kinetics of membrane depolarization are shown in Figures 9F–I. The bar graphs show the AUC of the kinetics of membrane depolarization when neutrophils were stimulated with (G) PMA, (H) L. monocytogenes, or (I) L. monocytogenes plus 2-APB or Xestospongin C. The graphs show the mean ± SD (n = 3). The statistical analysis was performed by Welch's t-test (G,H) or with ANOVA one-way and Sidak multiple comparisons (****p < 0.0001). [file Image_3.TIFF]
